# Evaluating the implementation of intermittent versus continuous fetal heart rate monitoring on intrapartum care and early neonatal outcomes using causal mediational modeling

**DOI:** 10.3389/fdsfr.2026.1755487

**Published:** 2026-06-09

**Authors:** Omkar Basnet, Rejina Gurung, Pratiksha Bhattarai, Merlind Maria Hofert, Sunil Mani Pokharel, Prajwal Paudel, Ashish K. C.

**Affiliations:** 1 Research Division, Golden Community, Lalitpur, Nepal; 2 Department of Women’s and Children’s Health, Uppsala University, Uppsala, Sweden; 3 School of Public Health and Community Medicine, Institute of Medicine, University of Gothenburg, Gothenburg, Sweden; 4 Department of Obstetrics and Gynecology, Bharatpur Hospital, Ministry of Health, Chitwan, Nepal; 5 Department of Pediatrics, Paropakar Maternity and Women’s Hospital, Kathmandu, Nepal

**Keywords:** continuous vs. intermittent monitoring, fetal heart rate monitoring, implementation research, neonatal resuscitation, Nepal

## Abstract

**Introduction:**

Intrapartum fetal heart rate (FHR) monitoring can detect fetal compromise and provide indications for early action. However, the effect of implementing intermittent vs. continuous FHR monitoring with Moyo remains unclear. In this study, we compare the effect of implementing intermittent vs. continuous FHR monitoring using Moyo on intrapartum care and neonatal outcomes.

**Methods:**

A prospective observational design was used to evaluate two different FHR monitoring methods in seven hospitals in Nepal, with intermittent monitoring as the control group and continuous monitoring as the intervention group. The durations from 1) first Moyo placement to abnormal FHR detection and 2) first abnormal FHR detection to childbirth were compared using generalized linear regression. The causal mediational method assessed the risk of non-crying neonates after birth and intrapartum stillbirth between the groups using the Mantel–Haenszel (M–H) homogeneity method, standardizing the risk for mediators and confounders.

**Results:**

A total of 1,091 participants were enrolled during the control period and 8,687 during the intervention period. Compared to the control group, the intervention group was associated with a non-significant reduction in time from first Moyo placement to abnormal FHR monitoring [adj.β = −35·5 min; 95% confidence interval (CI) = −73·2 to 2·22] and a significant reduction in time from first abnormal FHR to delivery (adj.β = −66·5 min; 95% CI = −106·2 to −26·8). The incidence of non-crying neonates was higher in the control group (11·5% vs. 10·0%), with an adjusted risk reduction of 31% (M–H RR = 0·69; 95% CI = 0·55–0·87) in the intervention group. The incidence of intrapartum stillbirth was non-significantly higher in the control group (4.5 vs. 3.0/1,000 births), with an adjusted risk reduced by 35% (M–H RR = 0·65; 95% CI = 0·25–1·70) in the intervention group.

**Conclusion:**

Compared with intermittent Moyo, the duration to detect abnormal FHR and the time from abnormal FHR to delivery were significantly reduced, with a 31% reduction in the risk of neonatal resuscitation with continuous Moyo. The implementation of continuous FHR monitoring with portal devices such as Moyo can reduce the time to action following the detection of abnormal fetal heart rates and decrease the burden of infants requiring neonatal resuscitation.

**Clinical Trial Registration:**

clinicaltrials.gov, identifier ISRCTN16741720.

## Introduction

In South Asia, an estimated 34.7 million women gave birth in 2024, with nearly a million of them having intrapartum stillbirth and neonatal death (Amouzou et al., 2025). Despite the increase in the proportion of institutional births from 9% in 2001 to 78% in 2024, only one-third of women receive high-quality intrapartum care (Amouzou et al., 2025; Marthias et al., 2025). Poor quality of intrapartum care has been attributed to almost six million deaths over the past 25 years (Nove et al., 2021). Therefore, high-quality intrapartum care is vital to ensure that high-risk labor is identified early and intervened with for healthy outcomes for both mothers and newborns.

Intrapartum fetal heart rate (FHR) monitoring provides fetal surveillance of any intrapartum insults and indicates the need for intervention (Chandraharan et al., 2023; Richmond et al., 2025). However, barriers to effective FHR monitoring exist (Ekblom et al., 2022; Kc et al., 2024). In contrast to high-resource healthcare facilities where continuous FHR monitoring is standard, low-resource settings such as Nepal, where approximately 80% of births occur, rely on more affordable and practical methods such as handheld Doppler FHR detectors, the Pinard stethoscope, and strap-on electronic FHR monitors (Moyo) (KC et al., 2020; KC et al., 2016). The disproportionate increase in institutional births in low-resource facilities has not been matched by an adequate number of healthcare providers, resulting in persistently poor and even deteriorating quality of intrapartum care (KC et al., 2020; Khatri et al., 2022).

Although continuous FHR monitoring can provide more robust information on fetal heart rate tracing than intermittent FHR monitoring, there is limited evidence on whether continuous FHR monitoring can help healthcare providers initiate earlier interventions and reduce the time between the detection of an abnormal fetal heart rate and intervention (World Health Organization, 2018; Intrapartum, 2019; Alfirevic et al., 2006). Most importantly, the availability of portal FHR monitoring devices that not only monitor fetal heart rate but also provide real-time alarms to alert healthcare providers for intervention is essential (Musaba et al., 2025). Recent progress in FHR monitoring technology, such as the Moyo fetal heart rate monitor, provides both visual and audio signals on fetal heart rate status and progress (Laerdal Global Health, 2017). Moyo (Laerdal Global Health) is a portable FHR monitor that can display the fetal heart rate through sound wave signals, numerical displays, and color coding to indicate fetal heart rate status and trend, as well as maternal heart rates.

Using Moyo, we aimed to evaluate the implementation of intermittent vs. continuous FHR monitoring on the detection of abnormal FHR, time from first abnormal FHR detection to delivery, and change in early neonatal outcomes.

## Methods

*Study design:* This study is a secondary analysis of data collected within a previously registered clinical trial. This took place in seven public referral hospitals in Nepal over an 8-month period from 31 October 2020 to 29 June 2021, including 32,269 participants. Although the original trial was designed as a prospective cohort study, the present analysis uses an observational design comparing the periods of intermittent and continuous fetal heart rate monitoring. The exposure in the current analysis was not randomized, and all analyses were conducted accordingly.

*Study site:* The study was conducted in seven referral hospitals, which recorded 56,787 childbirths in 2022. The intrapartum stillbirth rates in these hospitals during the study period ranged 0·2%–0·8% (Supplementary Table 2). All participating hospitals are public referral centers, each representing one of Nepal’s provinces, where portable fetal heart rate monitors (Moyo) have been implemented. Each facility provides comprehensive obstetric and emergency care services.

*Study participants:* Women who were admitted to the labor unit, either not yet in labor or in the first stage of labor, were eligible for inclusion. Those who consented to the study were enrolled in the study. Women were excluded if, at the time of admission, they presented with stillbirth, fetal congenital malformation, estimated gestational age less than 22 weeks, were critically ill, or had an undetectable FHR and those women who were eligible but had no Moyo placements.

*Intervention:* The study period was divided into a control and an intervention period, each utilizing a single-type FHR monitoring. *Control period:* During the control phase, intermittent FHR monitoring was implemented across all participating hospitals as part of the standard care for Safer Births Bundle quality improvement initiative (Gurung et al., 2019). Under the guidance of the heads of obstetrics and nursing units, healthcare providers were trained to use the Moyo fetal rate monitor every 30 min during the first stage of labor and every 15 min during the second stage. Providers were trained to take immediate action if abnormal fetal heart rate readings persisted for 15 min or longer. For all women enrolled during this phase, continuous monitoring with the Moyo device was conducted until the time of delivery. All other procedures of fetal and infant care were similar to those in the control phase.

*Primary outcomes*: The primary outcomes were the time from first Moyo placement to abnormal FHR detection and the time from first abnormal FHR detection to delivery. *Secondary outcomes*: The secondary outcomes included 1) the time from first Moyo placement to delivery, 2) the time from last FHR check to delivery, 3) non-crying at birth, 4) need for neonatal ventilation, and 5) intrapartum stillbirth. *Co-variates (confounders and mediators):* Obstetric characteristics included maternal age, maternal and fetal complications at the time of admission, augmentation during labor and abnormal FHR during labor, and intrauterine resuscitation (Table 1 for definition).

**TABLE 1:** Operational definition of outcomes and co-variates.

Primary outcome	Definition
Time from first Moyo placement to abnormal FHR detection (minutes)	Duration from first placement of Moyo during labor until the detection of abnormal FHR (>110 < 160 bpm) for the first time. It was measured in minutes
Time from first abnormal FHR detection to delivery (minutes)	Duration from first detection of abnormal FHR (>110 < 160 bpm) until childbirth (delivery of shoulder). It was measured in minutes
Secondary outcome	
Time from first Moyo placement to delivery (minutes)	Duration of first placement of Moyo during labor until childbirth (delivery of shoulder). It is measured in minutes
Time from last FHR check to delivery (minutes)	Duration of the last fetal heart rate monitored during labor until childbirth (delivery of shoulder). It was measured in minutes
Non-crying at birth	Clinical sign of not crying after 15 s of birth who require neonatal resuscitation
Neonatal resuscitation	Any intervention (stimulation or ventilation) after birth to help transition into the non-crying or non-birthing infants
Neonatal ventilation	Non-crying infants after birth who were ventilated using bag and mask ventilation
Intrapartum stillbirth	Infants with no breathing or heart rate from birth until 10 min after birth
Co-variates	
Intrapartum complication	Any maternal or fetal complication during labor (intrapartum), such as prolonged labor, premature rupture of the membrane, pre-eclampsia, uterine cord prolapses, and infection
Intrapartum resuscitation	A set of events during intrapartum period, when fetal distress is identified, such as repositioning the mother, stopping uterine stimulants (oxytocin), administering intravenous fluid boluses, and using tocolytics to decrease uterine activity

*Data collection:* For this study, a validated, specially designed Moyo placement and monitoring clinical observation checklist, which included information on Moyo placement and FHR monitoring, was used to observe the labor and delivery event for all vaginal births. Obstetric and neonatal information was additionally collected from patient charts. The data collection system was set up at each participating hospital, and observations were made by independent clinical research officers. Data collection was conducted using a mobile-based web application, “SUSTAIN,” which was developed by the research team to facilitate efficient and accurate collection of both observational data and patient chart information in real time. The SUSTAIN application was designed to be user-friendly and could be easily operated on tablets or mobile devices, making it highly suitable for clinical settings. Key features of the application included patient registration, real-time data synchronization, and automatic time stamping of entries. It allowed the assignment of multiple forms to individual patients based on specific requirements, with real-time tracking and synchronization of completed forms. To ensure data quality, the application incorporated validation rules and skipped logic to minimize errors, while a color-coded system provided clear visual cues for completed, partially completed, or pending information. Overall, SUSTAIN application streamlined data collection, enhanced accuracy, and enabled efficient monitoring of study processes.

*Sample size:* This study was part of the quality improvement study powered to detect a change in intrapartum mortality. Using the *post hoc* analysis, a sample size of 590 participants with intrapartum fetal heart monitoring performed using each method was required to detect a 10% difference in mean duration (150 ± 60 min) between the groups with 80% statistical power and 95% level of significance.

*Data management and analysis:* Data were entered into a tablet-based application and reviewed on a weekly basis by an independent database manager. For this study, data were extracted into SPSS software (IBM SPSS Statistics for Windows, Version 23·0) and cleaned using MATLAB (version 2021b). Data consistency was checked, and mismatched cases were retrieved and corrected accordingly before the analysis. Pearson’s chi-square tests were used to test proportional differences. Independent sample t-tests were used to compare mean and standard deviation (SD). For continuous variables, skewness and normality tests were conducted. Due to persistent skewness, which remained even after log transformation, medians and interquartile ranges (25th, 50th, and 75th percentiles) were reported using centile analysis stratified by intervention type. Data cleaning involved the application of multiple imputation techniques to address missing values and extreme time measurements using the multiple imputation estimation (mi) in Stata.

Observations with negative time values were excluded from the analysis. Generalized linear model (GLM) analyses were conducted in Stata (version 18) for each log-transformed time interval separately, to compare outcomes between the continuous and intermittent monitoring groups.

To assess the robustness of our findings given the substantial imbalance between exposure groups and baseline differences, we conducted sensitivity analyses using propensity score-based methods. Propensity scores were estimated using logistic regression, including available key sociodemographic, health-related, and contextual covariates measured prior to exposure.

First, inverse probability weighting (IPW) was applied to create a weighted pseudo-population, in which baseline covariates were balanced between groups. Weighted regression models with robust standard errors were then estimated. Results from these analyses were compared with those of the primary models to evaluate the consistency of findings.

**FIGURE 1:**
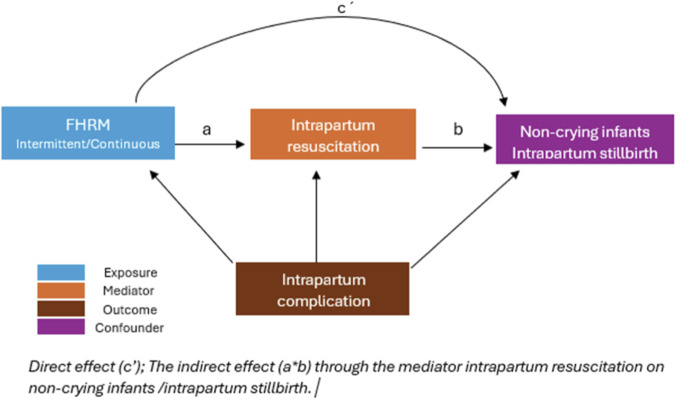
Directed acyclic graph (DAG) illustrating the mediator and confounder.

To assess differences across hospitals, meta-analyses were plotted in R (version 4·4.3) using RStudio (2024·12·1). For this purpose, GLMs were first run individually for each hospital using Stata, adjusting for relevant covariates including maternal and fetal complications, along with intrauterine resuscitation. A log link function and Gaussian distribution were adopted in the model. The resulting odds ratios were manually extracted and compiled into an Excel spreadsheet, which served as the input for generating meta plots in R. A directed acyclic graph (DAG) was constructed to assess the direct effect of the intervention through intrauterine resuscitation and non-crying among infants (Figure 1), and based on the distribution of the co-variates between the control and intervention groups in Supplementary Table 3, intrapartum resuscitation was considered a mediator, and intrapartum complication was considered a confounder. To evaluate this, we used the Mantel–Haenszel method.

*Ethics:* Ethical approval for this study was obtained from the National Ethical Review Board, Nepal Health Research Council. Ethical clearance was also received from the Institutional Review Board (IRB) of all participating hospitals. Written informed consent was taken from all participating women prior to data extraction and clinical observations.

## Results

As part of the quality improvement intervention, among the 32,269 deliveries that took place during the study period, 28,608 were eligible. Among them, 16,635 women were not monitored using the Moyo device, and 753 women did not provide consent for enrollment. Ultimately, 11,220 consented for observation; however, 1,442 of them were excluded as the use of continuous or intermittent Moyo was not in accordance with the protocol. The total observed cases were 9,778, with 1,091 in the control and 8,687 in the intervention group (Supplementary Figure 1). Results from both IPW and propensity score-matched analyses were consistent with the primary findings in terms of direction and magnitude of associations, although effect estimates were attenuated in some models. This consistency increases confidence that the observed associations are not solely driven by baseline imbalances between groups.

Among the demographic and obstetric characteristics, gestational age, the proportion of women with abnormal FHR detected during labor, and mode of delivery were similar between the groups. Between the groups, there was difference in admission complications (*p* < 0·001), intrapartum maternal complication (*p* < 0·0001), and place of birth (*p* < 0·0001) (Supplementary Table 3).

The median time from the first occurrence of abnormal FHR detection to delivery was 132 min [interquartile range (IQR): 55–242] in the control group compared with 73 min (IQR: 37–146) in the intervention group, with an unadjusted coefficient of −70.2 (95% confidence interval [CI] = −109.9 to −30.6). After adjusting for maternal and fetal complications during labor and intrauterine resuscitation, there was a 30% reduction in the time from the first abnormal FHR detection to delivery in the continuous group compared with the intermittent group [adjusted odds ratio (aOR): 0·70; 95% CI: 0.54–0.92; *p* = 0·01]. The duration from the last detection of abnormal FHR detection to delivery was reduced by 44.5 min after the implementation of continuous Moyo (unadjusted coefficient: −44.5; 95% CI: −83.1 to −5.9). After adjustment, there was 19% reduction in the time from the last abnormal FHR detection to delivery in the continuous group compared with the intermittent group (aOR: 0·81; 95% CI: 0.59–1.12; *p*-value = 0.204), without statistical significance. The median time from the first Moyo placement to delivery was 143 min (IQR: 75–253) in the continuous Moyo group and 92 min (IQR: 43–178) in the intermittent group, with an unadjusted coefficient of −67.0 (95% CI: −77.0 to −57.0). After adjusting for maternal and fetal complications during labor and intrauterine resuscitation, the time from the first Moyo placement to delivery was reduced by 38% in the continuous group compared with the intermittent group (aOR: 0·62; 95% CI: 0.59–0.66; *p*-value <0.0001) (Table 2).

**TABLE 2:** Comparison of time intervals (in minutes) between the control and intervention periods.

Indicator’s	Median (IQR)	Unadjusted co-efficient (minutes)*	Adjusted co-efficient (minutes)**	aOR (95% CI)***
First placement to abnormal detection (n = 631)				
Control (n = 61)	90.0 (44.0–241.5)	Reference	​	​
Intervention (n = 570)	90.0 (39.6–195.7)	−36.9 (−74.5 to 0.61), *p* = 0.054	−35.5 (−73.2 to 2.22), *p* = 0.065	0.79 (0.53–1.28), *p* = 0.247
First abnormal FHR detection to delivery (n = 630)				
Control (n = 61)	132.2 (55.0–242.4)	​	​	​
Intervention (n = 569)	73.0 (36.8–145.5)	−70.2 (−109.9 to −30.6), *p* = 0.001	−66.5 (−106.2 to −26.8) *p* = 0.001	0.70 (0.54–0.92), *p* = 0.010
Last abnormal FHR check to delivery (n = 630)				
Control (n = 61)	93.0 (30.3–211.2)	​	​	​
Intervention (n = 569)	64.0 (26.5–123.9)	−44.5 (−83.06 to −5.90), *p* = 0.024	−43.2 (−82.0 to −4.50), *p* = 0.029	0.81 (0.59–1.12), *p* = 0.204
First Moyo placement to delivery (n = 9,764)				
Control (n = 1,082)	143.2 (75.0–253.3)	​	​	​
Intervention (n = 8,682)	92.0 (43.2–177.8)	−67.0 (−77.0 to −57.0), *p* < 0·0001	−67.0 (−76.9 to −57.0), *p* < 0.0001	0.62 (0.59–0.66), *p* < 0.0001
Last FHR check to delivery (n = 9,765)				
Control (n = 1,086)	27.8 (11.9–72.3)	​	​	​
Intervention (n = 8,679)	21.7 (11.0–37.4)	−25.0 (−30.6 to −19.4), *p* < 0.0001	−25.1 (−30.7 to −19.5), *p* < 0.0001	0.58 (0.53–0.63), *p* < 0.0001

Abbreviations: IQR, inter-quartile range; aOR, adjusted odds ratio; FHR, fetal heart rate.

*GLM with case exposure, **GLM adjusted to maternal complication and intrapartum resuscitation with hospital as exposure, ***GLM with log link adjusted to maternal complication and intrapartum resuscitation with hospital as exposure.

**FIGURE 2:**
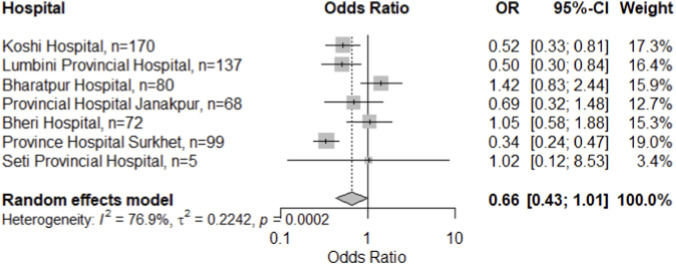
Forest plot on odds ratio of the first Moyo placement to the first FHR abnormal detection across hospitals, adjusted by intrapartum complications and intrauterine resuscitation (n = 631).

Meta-regression was conducted, expressed in a forest plot, to assess the odds of the first Moyo placement to the first FHR abnormality, which showed higher heterogeneity (τ^2^ value = 0·224) by hospital. The random effects model (REM) in the meta-regression showed 34% lower odds of the first placement of Moyo to the first FHR abnormality during intervention compared to the control period (REM, OR = 0.66; 95% CI = 0.43–1.01) (Figure 2).

**FIGURE 3:**
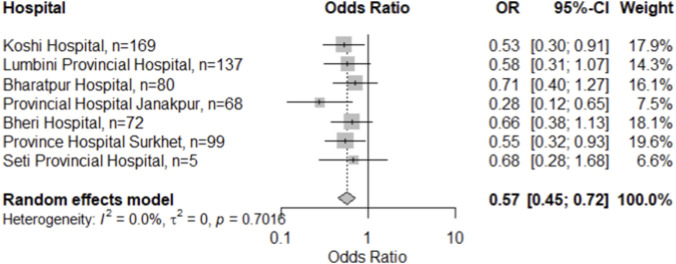
Forest plot on odds ratio of the first abnormal FHR detection to delivery across hospitals between the groups, adjusted by intrapartum complications and intrauterine resuscitation (n = 630).

The odds ratio of the first abnormal FHR detection to delivery had low heterogeneity (τ^2^ value = 0·0) by hospital between the study groups. The REM in meta regression showed significantly lower odds of 43% of the first placement of Moyo to the first FHR abnormality during intervention compared to the control group (REM, OR = 0.57; 95% CI = 0.45–0.72) (Figure 3).

Using meta-regression, the odds ratio for the duration from the last abnormal FHR detection to delivery showed higher heterogeneity (τ^2^ value = 0.274) between hospitals across the study periods. Meta-regression showed lower odds of 48% of the last abnormal FHR detection to delivery during intervention compared to the control period (REM, OR = 0.52; 95% CI = 0.31–0.86) (Supplementary Figure 1). Using the forest plot, the odds ratio of the first Moyo placement to delivery had low heterogeneity (τ^2^ = 0·094) between hospitals across the study periods. Meta-regression showed lower odds of 34% of the first Moyo placement to delivery during intervention compared to the control period (REM, OR = 0.66; 95% CI = 0.52–0.83) (Supplementary Figure 2). Using the forest plot, the odds ratio of the last FHR check to delivery had low heterogeneity (τ^2^ = 0.039) between hospitals across the study periods. Meta-regression showed lower odds of 42% of the first Moyo placement to delivery during intervention compared to the control period (REM, OR = 0.58; 95% CI: 0.48–0.70) (Supplementary Figure 3).

The incidence of non-crying infants after birth was higher during the control period than during the intervention period (11.5% vs. 10.0%). Using the mediational analysis, with intrapartum resuscitation as the mediator and intrapartum complication as the confounder, the risk of non-crying infants was 31% lower in the intervention period, with a statistical significance [M–H homogeneity RR: 0.69; 95% CI: 0.69 (0.55–0.87)] (Table 3).

**TABLE 3:** Non-crying infants before and after intervention, mediational analysis using the Mantel–Haenszel (M–H) method.

Indicator’s	Incidence rate	Risk ratio (95% CI)	Attributable fraction among the population
Non-crying among infants after birth among the control group (125/1,091)	11.5%	References	​
Non-crying among infants after birth among the intervention group (864/8,687)	10.0%	0.86 (0.73–1.04)	12.0%

*Intrapartum resuscitation as the mediator and intrapartum complication as the confounder using the Mantel*–*Haenszel (M*–*H) method*			
Mediational analysis		Risk ratio (95% CI)	M–H weight
Women with no intrapartum complication and no intrapartum resuscitation		0.69 (0.53–0.90)	49·4%
Women with no intrapartum complication and intrapartum resuscitation		0.89 (0.39–2.01)	4·6%
Women with intrapartum complication and no intrapartum resuscitation		0.48 (0.25–0.93)	6·78%
Women with intrapartum complication and intrapartum resuscitation		0.85 (0·23–3.1)	1·71%
Crude		0.87 (0.73–1.04)	​
M–H combined		0.69 (0.55–0.87)	​
Test of homogeneity (M–H)		chi^2^(3) = 1.63	*p* = 0.6519

The incidence of neonatal ventilation was 5·1% during the control period compared with 4·3% during the intervention period, with a 15% lower crude risk in the intervention group (cRR: 0.85; 95% CI: 0.64–1.11). In the mediational analysis, the risk of ventilation was 31% lower in the intervention period, which was statistically significant [M–H homogeneity RR: 0.69; 95% CI: 0.69 (0.48–0.99)] (Supplementary Table 4).

The rate of intrapartum stillbirth during the control period was 4·5 per 1,000 births compared with 3.0 per 1,000 births during the intervention period, corresponding to a non-significant 35% lower crude risk in the intervention group [cRR, 95% CI: 0.65 (0.25–1.70]. Using the mediational analysis, the risk of intrapartum stillbirth was 46% lower in the intervention period (M–H homogeneity RR: 0.54; 95% CI: 0.17–1.77), without statistical significance (Supplementary Table 5).

## Discussion

This observational study showed that compared to intermittent Moyo, continuous Moyo use during labor reduced the duration of detecting abnormal FHR monitoring by 37 min, the duration of the first FHR detection to delivery by 66.5 min, the last abnormal FHR check to delivery by 43 min, the first Moyo placement for FHR measurement to delivery by 67 min, and the last FHR check to delivery by 25 min. Interpretation of the time to the first abnormal fetal heart rate detection should be made cautiously as monitoring frequency differed between continuous and intermittent monitoring periods as per study design. The heterogeneity of the result existed among the hospitals; however, the adjusted meta-odds of the first Moyo placement to delivery were reduced by 43% when using continuous Moyo vs. intermittent Moyo. Similarly, the meta-odds of the last abnormal FHR detection to delivery were reduced by 48% when using continuous vs. intermittent Moyo. Using the causal mediational analysis, the risk of non-crying infants and resuscitation were reduced by 31% when using continuous Moyo vs. intermittent Moyo. However, there was no significant difference in the intrapartum stillbirth rate between continuous and intermittent Moyo. No statistically significant association was observed between intervention and stillbirths. Given the low incidence of stillbirths in the study population, the analysis may have been underpowered to detect modest differences between periods. Therefore, the absence of a statistically significant effect should not be interpreted as evidence of no effect, but rather as reflecting limited statistical power and the rarity of the outcome.

The uniqueness of the study is that it assessed the different intrapartum care and outcome comparing continuous *versus* intermittent monitoring with Moyo in Nepal. Most previous studies evaluated adverse delivery outcomes by comparing continuous *versus* intermittent monitoring using two different devices, such as handheld Dopplers *versus* electronic monitoring devices (Vintzileos et al., 1993; Mahomed et al., 1992) or Dopplers *versus* Fetoscopes (Mdoe et al., 2018a), or using cardiotocography measurements (Alfirevic et al., 2006). The latter could also not find evidence for an improvement in perinatal death, while most other studies, which have been designed as a randomized controlled trial, found reduced mortal neonatal outcomes (Alfirevic et al., 2006; Vintzileos et al., 1995; Thacker et al., 2001). This aligns well with our study outcomes and provides strong supporting evidence. A plausible explanation for the observed outcomes is that prolonged hypoxic events may occur when intervention is delayed in response to FHR abnormalities. This highlights the value of early detection through continuous monitoring. When examining the heterogeneity of study outcomes across the seven participating hospitals, the overall effect size indicates a shorter time to detection of abnormalities with continuous monitoring compared to intermittent monitoring. Although some consistency is observed across hospitals depending on the specific time intervals assessed, variability emerges in the key variable measuring the time from the initial Moyo placement to the first detection. This heterogeneity may be driven by hospital-specific factors such as clinical protocols, staff responsiveness, and monitoring infrastructure.

Several measures were implemented to minimize assessment bias, although blinding of care providers and participants was not feasible due to the nature of fetal heart rate monitoring modalities. Outcome definitions were pre-specified and based on standardized clinical criteria, and data collection followed a structured protocol to ensure consistent assessment across study groups. Clinical staff involved in monitoring and outcome recording received training in the interpretation of fetal heart rate patterns and study procedures to reduce variability in assessment.

Where possible, outcomes were derived from routinely collected clinical records rather than subjective judgment, reducing the risk of differential outcome assessment between continuous and intermittent monitoring groups. In addition, sensitivity analyses using propensity score-based methods were conducted to assess the robustness of findings to baseline imbalances between the groups. Both groups showed propensity scores across a similar range. Nevertheless, some degree of assessment bias cannot be fully excluded, particularly for outcomes that rely on clinical interpretation or decision-making, and this should be considered when interpreting the results.

## Implementation context

This was a hospital-based study in district and regional hospitals to evaluate the effectiveness of continuous FHR monitoring during labor. The seven participating hospitals varied in terms of equipment, annual delivery volume, human resources, and infrastructure, enhancing the generalizability of the findings. Each hospital’s labor unit was staffed by skilled birth attendants and equipped with neonatal resuscitation services at the time of birth. Training on the correct use of the Moyo device was conducted prior to the intervention period as poor adherence beforehand had potentially negatively impacted perinatal mortality rates. The Moyo device, a digital FHR monitor, uses a highly accurate 9-crystal Doppler ultrasound sensor, which can detect FHR accurately. The visual and audio alerts promote timely intervention (Laerdal Global Health). The timing of the training may have influenced the level of preparedness among healthcare professionals prior to the intervention, potentially contributing to improved performance during the intervention period compared to the control period. This indicates residual measurement heterogeneity.

High-volume hospitals such as Bharatpur, Lumbini, Koshi, and Janakpur face significant shortages in human resources, with a single healthcare provider responsible for an average of 900 deliveries per year (Chaulagain et al., 2022). This workload suggests that, at any given time, there may be at least eight women in labor and two actively delivering in the same delivery room. Compared with a reported annual delivery of 272 per healthcare worker in China (Zhu et al., 2024), the number in several hospitals in Nepal seems to be extremely high, likely compromising the quality of neonatal care. Under such conditions, conducting intermittent FHR monitoring with a time interval of 30 min would become difficult. Continuous FHR with an integrated alarm system offers a convenient and practical alternative for routine use. More specifically, it improves the midwife’s ability of detecting abnormal FHR in two ways: first, by providing visual documentation of abnormalities through a 30-min histogram review of the tracing; and second, by triggering an alarm if an abnormal FHR persists for 3 minutes. This latter feature allows midwives to monitor multiple mothers simultaneously, which is a major advantage of this device. These translated into improved FHR monitoring practices including timely responses, such as reduced time to the detection of an abnormal FHR following admission, shorter times from the last FHR measurement to delivery, and shorter overall duration of labor.

### Generalizability

Meta-regression analyses demonstrated consistent associations across hospitals, supporting the robustness of findings across settings. In addition, the findings of the study are similar with those conducted in Tanzania where detection of abnormal FHR was higher with continuous Moyo than in intermittent Doppler assessments (). Another study in Tanzania showed that the use of continuous FHR monitoring with Moyo not only increased the detection of abnormal FHR but also led to more frequent intrauterine resuscitations (Mdoe et al., 2018b). Similar to our study results, they show significantly fewer cases of resuscitations in the continuous Moyo group than in the intermittent group. Mahomed et al. observed the best neonatal outcomes in the group of continuous monitoring. Even though these studies used different devices, the results lead to the same direction toward a causal relationship between the type of monitoring and the birth outcome.

The time from abnormal FHR detection to delivery was shorter in the continuous Moyo than in the intermittent Moyo. The study in Tanzania shows similarity to our findings with abnormal FHR detection earlier in Moyo compared to Doppler (Mdoe et al., 2018a). One of the studies showed that the time interval from the first abnormal FHR detection to birth was comparable between Pinard fetoscope and Doppler, while another showed that it was significantly longer with Moyo than with Doppler (Kamala et al., 2019). However, in our study, detection of abnormal FHR has prompted action with significantly shorter time from abnormal FHR detection to childbirth.

## Methodological consideration

Although time-to-event data are often right-skewed, the observed distribution in this study was approximately symmetric, supporting the use of Gaussian models. The use of causal mediational methods to standardize the risk for the mediator and confounder is the strength of the study. Given the susceptibility of observational studies, our study findings are subject to unmeasured confounding and may reflect selection bias. The high uptake of continuous Moyo monitoring may also reflect the support provided by research staff who guided and observed the QI process in the hospitals. Additionally, there may have been some errors in recording time intervals during data collection. When interpreting the results, it should be considered that neonatal mortality was not reflected in the outcomes, unlike in other studies. At least, the proportionally small control group makes it more difficult to observe significant differences compared to the intervention group.

Several measurement limitations should be acknowledged. Differences in monitoring modality inherently influence the frequency and timing of fetal heart rate assessment, which may affect detection-related outcomes. As a result, comparison of time-based outcomes between intermittent and continuous monitoring periods may be affected by differences in documentation practices and monitoring intensity, potentially limiting direct comparability. Additionally, reliance on routine clinical documentation may introduce variability in the recording of monitoring events and outcomes across settings. There was selection bias, where the participants who were eligible but not enrolled had differences in obstetric and background characteristics (Supplementary Table 6). However, characteristics were adjusted in the casual mediational analysis, and there was no variance in terms of enrollment by hospitals (Supplementary Table 7).

### Policy and practice implications

The widespread implementation of continuous FHR monitoring using portable devices such as Moyo has the potential to significantly reduce the time required to detect FHR abnormalities. This, in turn, can shorten the time to clinical action and help reduce the burden of stillbirth. Additionally, the lower risk for intrapartum resuscitation saves time resources and might lead to less health burden in further life. Compared to intermittent monitoring, continuous FHR monitoring with such devices also reduces the workload for healthcare providers and enhances the overall quality of intrapartum care. The easy implementation and the low acquisition costs make this intervention a cost-effective and sustainable opportunity to improve maternal and neonatal health. Given these benefits, investing in this type of monitoring technology is recommended as a strategy to promote healthy lives from an early stage.

## Data Availability

The raw data supporting the conclusions of this article will be made available by the authors, without undue reservation.
