# Supplementary material for: Evaluating the implementation of intermittent versus continuous fetal heart rate monitoring on intrapartum care and early neonatal outcomes using causal mediational modeling
Source: Front Drug Saf Regul. 2026 Jun 9;6:1755487. doi: 10.3389/fdsfr.2026.1755487 (PMC13287916; doi:10.3389/fdsfr.2026.1755487)
Supplement: Supplementary file 1 [file Table1.docx]

Supplementary file

**Evaluating implementation of intermittent versus continuous foetal heart rate monitoring on intrapartum care and birth outcome- using causal mediational modelling**

Contents

[Supplementary figure 1. Study flow of the participants 2](#_Toc212210082)

[***Supplementary figure 2****. Forest plot on odds ratio of last abnormal FHR detection to delivery across Hospitals between the groups, adjusted by intrapartum Complications and intrauterine resuscitation (n=630)* 3](#_Toc212210083)

[***Supplementary figure 3.*** *Forest plot on odds ratio of first MOYO Placement to delivery across Hospitals between the groups, adjusted by intrapartum complications and intrauterine resuscitation (n=9764)* 3](#_Toc212210084)

[**Supplementary figure 4.** Forest plot on odds ratio of last FHR Check to Delivery across Hospitals between the groups, adjusted by intrapartum complications and intrauterine resuscitation (n=9765) 4](#_Toc212210085)

[**Supplementary table 1.** Systematic literature search on fetal heart rate monitoring study in South East Asia 5](#_Toc212210086)

[***Supplementary table 2.*** *Stillbirth rate of study population in the observing hospitals* 6](#_Toc212210087)

[***Supplementary table 3.*** *Distribution of demographic and obstetric characteristics of the participants* 6](#_Toc212210088)

[***Supplementary table 4.*** *Ventilation before and after intervention, mediational analysis using Mantel-Haenszel (M-H) method* 7](#_Toc212210089)

[***Supplementary t*able *5*.** Intrapartum Stillbirth before and after intervention, mediational analysis using Mantel-Haenszel (M-H) method 7](#_Toc212210090)

# Supplementary figure 1. Study flow of the participants

Excluded

Excluded

Excluded

Did not complete FHRM (n=1442)

Did not consent (n=17388)

Total population enrolled to the study (n=11,220)

Total population eligible for the study (n=28,608)

Total population who delivered during the study period (n=32,269)

Total participants who completed FHRM as per protocol (n=9,778)

Did not meet the eligibility criteria (n=3661)

# ***Supplementary figure 2****. Forest plot on odds ratio of last abnormal FHR detection to delivery across Hospitals between the groups, adjusted by intrapartum Complications and intrauterine resuscitation (n=630)*


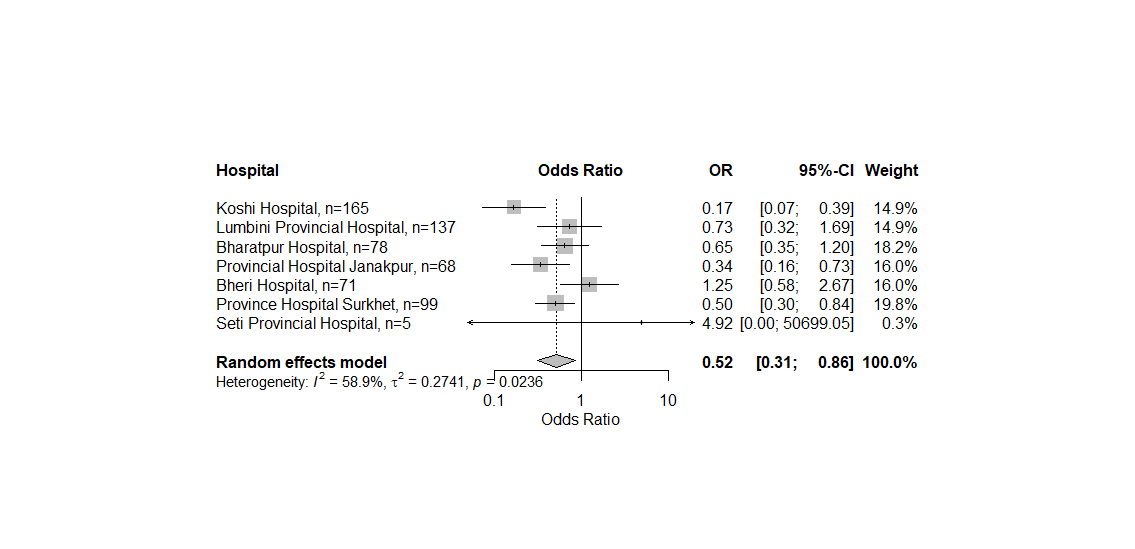


# ***Supplementary figure 3.*** *Forest plot on odds ratio of first MOYO Placement to delivery across Hospitals between the groups, adjusted by intrapartum complications and intrauterine resuscitation (n=9764)*


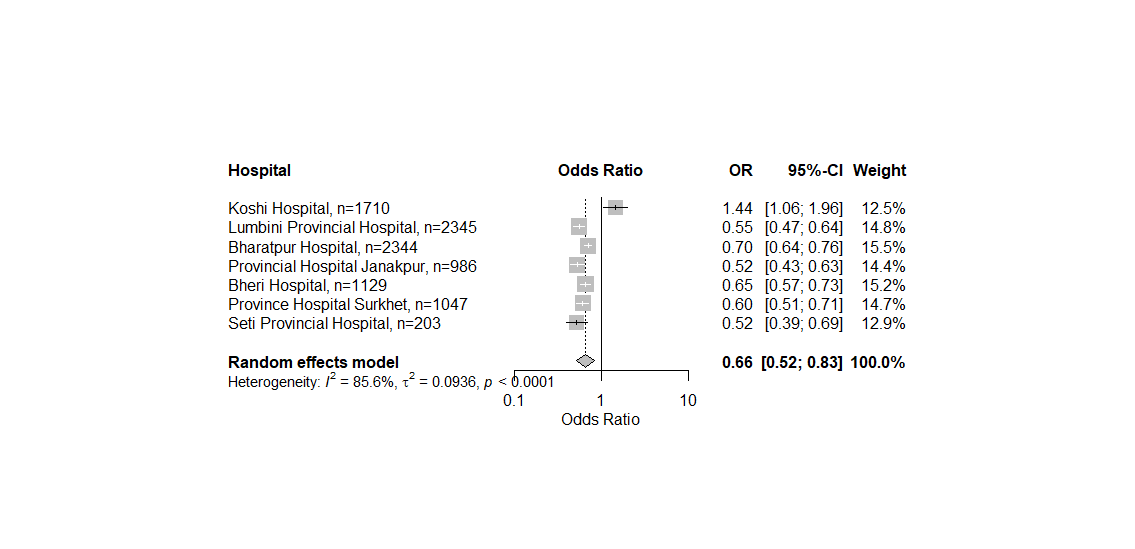


# **Supplementary figure 4.** Forest plot on odds ratio of last FHR Check to Delivery across Hospitals between the groups, adjusted by intrapartum complications and intrauterine resuscitation (n=9765)


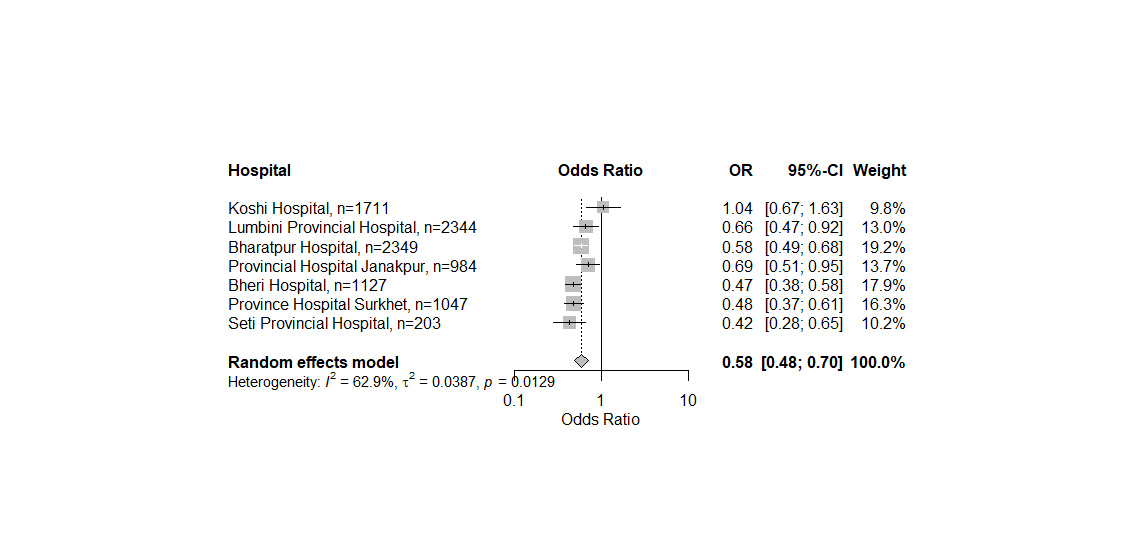


# **Supplementary table 1.** Systematic literature search on fetal heart rate monitoring study in South East Asia

| Search number | Query | Sort By | Search Details | | Results | Time | Date |  |
| --- | --- | --- | --- | --- | --- | --- | --- | --- |
| 4 | ("Intermittent Fetal Health rate monitoring") AND ("continuous fetal heart rate monitoring") | Most Recent |  | ("intermittent"[All Fields] OR "intermittence"[All Fields] OR "intermittencies"[All Fields] OR "intermittency"[All Fields] OR "intermittent"[All Fields] OR "intermittently"[All Fields]) AND ("fetale"[All Fields] OR "fetally"[All Fields] OR "fetals"[All Fields] OR "fetus"[MeSH Terms] OR "fetus"[All Fields] OR "fetal"[All Fields] OR "foetal"[All Fields]) AND ("health"[MeSH Terms] OR "health"[All Fields] OR "health s"[All Fields] OR "healthful"[All Fields] OR "healthfulness"[All Fields] OR "healths"[All Fields]) AND ("j rehabil assist technol eng"[Journal] OR "rate"[All Fields]) AND ("monitor"[All Fields] OR "monitor s"[All Fields] OR "monitorable"[All Fields] OR "monitored"[All Fields] OR "monitoring"[All Fields] OR "monitoring s"[All Fields] OR "monitorings"[All Fields] OR "monitorization"[All Fields] OR "monitorize"[All Fields] OR "monitorized"[All Fields] OR "monitors"[All Fields]) AND "continuous fetal heart rate monitoring"[All Fields] | 0 | 09:11:00 | 19/08/2025 | |
| 3 | ("Intermittent Fetal Health rate monitoring") AND ("continuous fetal heart rate monitoring") - Schema: all | Most Recent |  | ("Intermittent Fetal Health rate monitoring") AND ("continuous fetal heart rate monitoring") | 0 | 09:11:00 | 19/08/2025 | |
| 2 | "Intermittent Fetal Health rate monitoring" | Most Recent |  | ("intermittant"[All Fields] OR "intermittence"[All Fields] OR "intermittencies"[All Fields] OR "intermittency"[All Fields] OR "intermittent"[All Fields] OR "intermittently"[All Fields]) AND ("fetale"[All Fields] OR "fetally"[All Fields] OR "fetals"[All Fields] OR "fetus"[MeSH Terms] OR "fetus"[All Fields] OR "fetal"[All Fields] OR "foetal"[All Fields]) AND ("health"[MeSH Terms] OR "health"[All Fields] OR "health s"[All Fields] OR "healthful"[All Fields] OR "healthfulness"[All Fields] OR "healths"[All Fields]) AND ("j rehabil assist technol eng"[Journal] OR "rate"[All Fields]) AND ("monitor"[All Fields] OR "monitor s"[All Fields] OR "monitorable"[All Fields] OR "monitored"[All Fields] OR "monitoring"[All Fields] OR "monitoring s"[All Fields] OR "monitorings"[All Fields] OR "monitorization"[All Fields] OR "monitorize"[All Fields] OR "monitorized"[All Fields] OR "monitors"[All Fields]) | 98 | 09:10:12 | 19/08/2025 | |
| 1 | "continuous fetal heart rate monitoring" | Most Recent |  | "continuous fetal heart rate monitoring"[All Fields] | 63 | 09:09:41 | 19/08/2025 | |

# ***Supplementary table 2.*** *Stillbirth rate of study population in the observing hospitals*

|  | Total No. Births | Intrapartum Stillbirth Rate |
| --- | --- | --- |
| Koshi Hospital | 1,712 | 0·47 % |
| Lumbini Provincial Hospital | 2,345 | 0·17 % |
| Bharatpur Hospital | 2,351 | 0·43 % |
| Provincial Hospital Janakpur | 987 | 0·51 % |
| Bheri Hospital | 1,132 | 0·80 % |
| Province Hospital Surkhet | 1,048 | 0·29 % |
| Seti Provincial Hospital | 203 | 0·49 % |

# ***Supplementary table 3.*** *Distribution of demographic and obstetric characteristics of the participants*

|  | Control period (n=1,091) | Intervention period (n=8,687) | p-value* |
| --- | --- | --- | --- |
| Mother’s age in years (mean, SD) | 23·8±4·4 | 23·9±4·2 | 0·481 |
| Gestational age in weeks (mean, SD) | 39·6±6·5 | 38·9±3·9 | <0·001 |
| Admission complications |  |  |  |
| No | 1,022(93·7%) | 7,875 (90·7%) |  |
| Yes | 69 (6·3%) | 812 (9·3%) | 0·001 |
| Intrapartum complication (maternal) |  |  |  |
| No | 1,067 (97·8%) | 8,221 (94·6%) |  |
| Yes | 24 (2·4%) | 466 (5·4%) | <0·0001 |
| Intrapartum resuscitation |  |  |  |
| No | 1,050 (96·2%) | 8,388 (96·6%) |  |
| Yes | 41 (3·8%) | 299 (3·4%) | 0·591 |
| Intrapartum complication (fetal) |  |  |  |
| No | 1,046 (95·9%) | 8,341 (96·0%) |  |
| Yes | 45 (4·1%) | 346 (4·0 %) | 0·822 |
| Intrapartum complication (combined) |  |  |  |
| No | 1,022 (93·7%) | 7,875 (90·7%) |  |
| Yes | 69 (6·3%) | 812 (9·3%) | 0·001 |
| Labor augmentation |  |  |  |
| No | 610 (55·9%) | 4,625 (53·2%) |  |
| Yes | 481 (44·1%) | 4,062 (46·8%) | 0·10 |
| Delivery method |  |  | 0·861 |
| Vaginal | 910 (83·4%) | 7,300 (84·0%) |  |
| Instrumental | 88 (8·1%) | 667 (7·7%) |  |
| Emergency C-section | 93 (8·5%) | 720 (8·3%) |  |
| Hospitals |  |  | <0·0001 |
| Koshi | 91 (8·3%) | 1,621 (18·6%) |  |
| Lumbini | 64 (5·9%) | 2,281 (26·2%) |  |
| Bharatpur | 548 (50·2%) | 1,803 (20·8%) |  |
| Janakpur | 83 (7·6%) | 904 (10·4%) |  |
| Bheri | 233 (21·4%) | 899 (10·3%) |  |
| Surkhet | 57 (5·2%) | 991 (11·4%) |  |
| Seti | 15 (1·4%) | 188 (2·2%) |  |

*Abbreviations: SD=standard deviation; FHR=foetal heart rate*

# ***Supplementary table 4.*** *Ventilation before and after intervention, mediational analysis using Mantel-Haenszel (M-H) method*

|  | Incidence rate | Risk ratio (95% CI) | | Attributable fraction among population | |
| --- | --- | --- | --- | --- | --- |
| Ventilation among infants in the control group (56/1091) | 5·1% |  | |  | |
| Ventilation among infants in the intervention group (377/8687) | 4·3% | 0·85 (0·64, 1·11) | | 13·7% | |
| *Intrapartum resuscitation as medicator and intrapartum complication as confounder using Mantel-Haenszel (M-H) method* | | | | | |
| **Mediational analysis** | | | Risk ratio (95% CI) | | M-H weight |
| Women with no intrapartum complication and no intrapartum resuscitation | | | 0·71 (0·46, 1·09) | | 19·96% |
| Women with no intrapartum complication and intrapartum resuscitation | | | 1·78 (0·25, 12·7) | | 0·92% |
| Women with intrapartum complication and no intrapartum resuscitation | | | 0·33 (0·16, 0·68) | | 5·82% |
| Women with intrapartum complication and intrapartum resuscitation | | | 1·54 (0·23, 10·3) | | 0·85% |
| Crude | | | 0·85 (0·64, 1·11) | |  |
| M–H combined | | | 0·69 (0·48, 0·99) | |  |
| Test of homogeneity (M-H) | | | chi2(3) = 5·56 | | p=0·1349 |

# ***Supplementary t*able *5*.** Intrapartum Stillbirth before and after intervention, mediational analysis using Mantel-Haenszel (M-H) method

|  | Incidence rate | Risk ratio (95% CI) | | Attributable fraction among population | |
| --- | --- | --- | --- | --- | --- |
| Intrapartum stillbirth in the control group (5/1091) | 4·5/1000 birth (0·45%) |  | |  | |
| Intrapartum stillbirth in the intervention group (26/8687) | 3·0/1000 birth (0·30 %) | 0·65 (0·25, 1·70) | | 30·8% | |
| *Intrapartum resuscitation as medicator and intrapartum complication as confounder using Mantel-Haenszel (M-H) method* | | | | | |
| **Mediational analysis** | | | Risk ratio (95% CI) | | M-H weight |
| Women with no intrapartum complication and no intrapartum resuscitation | | | 0 | | 0 |
| Women with no intrapartum complication and intrapartum resuscitation | | | 0·18 (0·03, 0·92) | | 1·84% |
| Women with intrapartum complication and no intrapartum resuscitation | | | 0·28 (0·03, 2·12) | | 0·97% |
| Women with intrapartum complication and intrapartum resuscitation | | | 0 | | 0 |
| Crude | | | 0·65 (0·25, 1·70) | |  |
| M–H combined | | | 0·54 (0·17, 1·77) | |  |
| Test of homogeneity (M-H) | | | chi2(3) = 2·20 | | p=0·1384 |
